# Optimization of Parameter Selection for Partial Least Squares Model Development

**DOI:** 10.1038/srep11647

**Published:** 2015-07-13

**Authors:** Na Zhao, Zhi-sheng Wu, Qiao Zhang, Xin-yuan Shi, Qun Ma, Yan-jiang Qiao

**Affiliations:** 1 Beijing University of Chinese Medicine, Beijing 100102, China; 2 Beijing Key Laboratory for Basic and Development Research on Chinese Medicine, Beijing, 100102, China; 3 Key Laboratory of TCM-information Engineer of State Administration of TCM, Beijing, China, 100102

## Abstract

In multivariate calibration using a spectral dataset, it is difficult to optimize nonsystematic parameters in a quantitative model, i.e., spectral pretreatment, latent factors and variable selection. In this study, we describe a novel and systematic approach that uses a processing trajectory to select three parameters including different spectral pretreatments, variable importance in the projection (VIP) for variable selection and latent factors in the Partial Least-Square (PLS) model. The root mean square errors of calibration (RMSEC), the root mean square errors of prediction (RMSEP), the ratio of standard error of prediction to standard deviation (RPD), and the determination coefficient of calibration (R_cal_^2^) and validation (R_pre_^2^) were simultaneously assessed to optimize the best modeling path. We used three different near-infrared (NIR) datasets, which illustrated that there was more than one modeling path to ensure good modeling. The PLS model optimizes modeling parameters step-by-step, but the robust model described here demonstrates better efficiency than other published papers.

Near-infrared (NIR) Spectroscopy combined with Partial Least-Square (PLS) has gained wide acceptance because it is rapid, nondestructive, and environmentally friendly. It has been successfully applied to different fields including agriculture^1^,^2^, food stuffs^3^, petrochemistry^4^, and pharmacy^5^,^6^,^7^. In addition, NIR technology offers sufficient accuracy and precision for solid and liquid systems without any sample pretreatment.

However, establishing a reliable and robust PLS model is a vital procedure for successful application of NIR. There are many parameters that should be optimized in the PLS model. These include spectral pretreatment, variable selection and latent factors. The NIR spectra contain unexpected chemical interferences related to sampling error, instrument error, etc.

In the process of establishing a PLS model, spectral pretreatment methods have been applied to extract relevant information and reduce the effect of noise and baseline drift^8^,^9^. Variable selection is then also used to identify highly informative features and eliminate useless variables from the original spectral dataset^10^,^11^. The latent factors that explain the spectral matrix are sorted in decreasing order according to their contribution to the spectral features. It is crucial to determine the latent factors and avoid over-fitting or under-fitting^12^,^13^.

Most published work about PLS models offer few details about the model development. It is assumed that there is a univariate used to select these parameters one by one according the root mean square error of cross validation (RMSECV) and the root mean square errors of prediction (RMSEP). The inter-influence among the three parameters is rarely considered in model development. This is risky because the modeling path is not necessarily the best approach to step-by-step optimization.

Wenlong Li *et al*. established quantitative methods based on NIR to determine the active compounds of *Lonicerae Japonicae Flos*^14^. Ioan Tomuta *et al*. applied NIR for the determination of active pharmaceutical ingredients (API) and pharmaceutical properties including the crushing strength and disintegration time of meloxicam tablets^15^. Nádia Reis *et al*. developed a quantitative method for multiple adulterants in roasted coffee using diffuse reflectance infrared Fourier transform spectroscopy^16^. Yajie Xi *et al*. developed a NIR method to determinate the trace dimethyl fumarate in milk^17^. In the above reports, spectral pretreatment methods were compared according to the predictive performance of the calibration models. Using the optimal pretreatment method, latent factors were selected with a cross validation method. The optimum wavelength range was then tested and selected based on these pretreatment and latent variables. Although quantitative models could be obtained, they did not investigate the trajectory routes of PLS models that consider the combination of different parameters.

Based on this consideration, we propose a novel and systematic approach to improve the efficiency and accuracy in the development of PLS models. The tracking procedure of PLS modeling provided a systematic profile that combined different spectral pretreatment methods, latent factors, and the variable importance in the projection (VIP) variable selection. The model performance was assessed using the root mean square errors of calibration (RMSEC), RMSEP, the determination coefficient of calibration (R_cal_^2^) and validation (R_pre_^2^) as well as the ratio of standard error of prediction to standard deviation (RPD)^18^,^19^. To demonstrate the advantage of this novel approach, three different NIR spectral datasets were analyzed including two standard and open source datasets. The proposed procedure was used to predict water, baicalin, and API in corn, Yinhuang granules, and pharmaceutical tablets, respectively. These specific NIR applications were selected because they are important to quality control.

## Result

### Near-infrared spectral features

**Figure 1:**
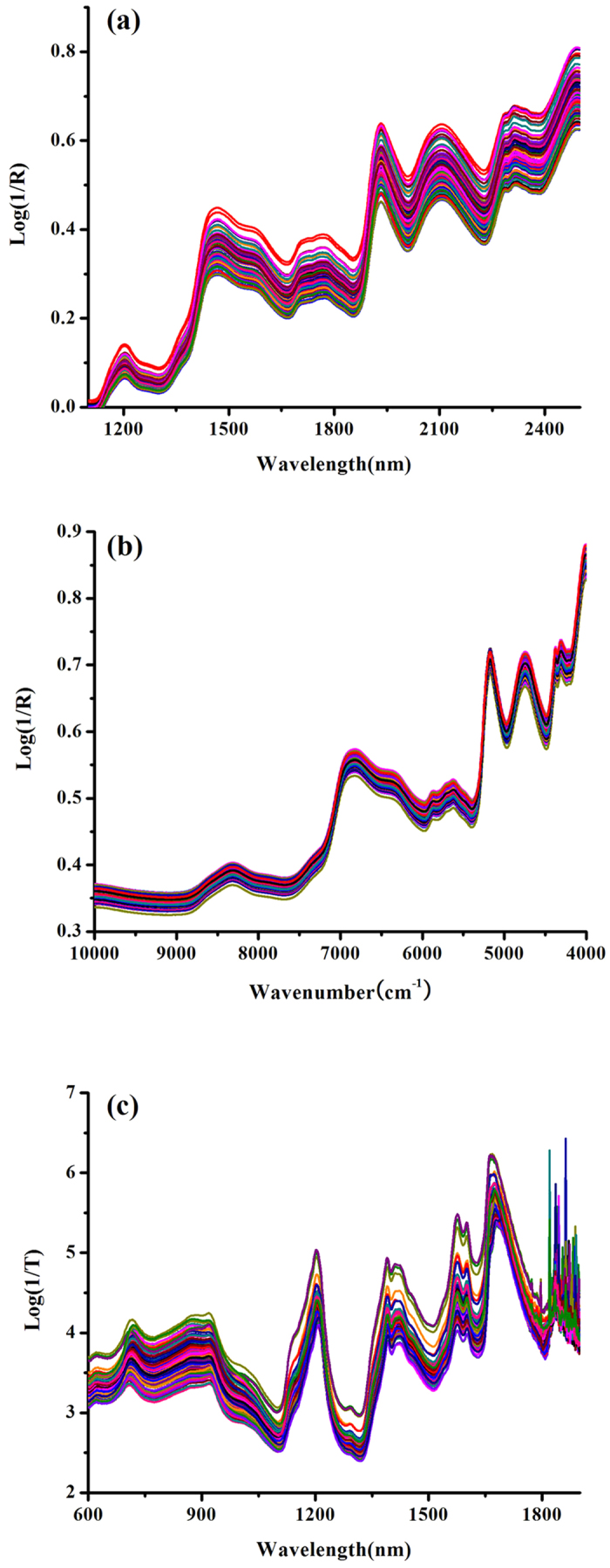
Raw NIR spectra of corn sample (**a**), Yinhuang granules sample (**b**) and pharmaceutical tablets sample (**c**).

The NIR spectra (1,100–2,498 nm) of typical corn samples are shown in Fig. 1a. There were several broad peaks located near 1,190, 1,450, and 1,940 nm. These represent the characteristic peaks regarding the water in the corn spectral dataset. The strong absorption bands of water appeared around 1,400–1,450 nm and 1,900–1,950 nm due to the first overtone of OH stretching and combination of the OH stretching band with OH bending.

On the other hand, the NIR spectra of Yinhuang granules are shown in Fig. 1b. There was a weak absorption in the second overtones region (SCOT, 10,000–7,100 cm^−1^) of the fundamental CH stretching bands. There was much fluctuation in the region of first combination-overtone (FCOT, 7,100–4,900 cm^−1^) and combination region (CR, 4,900–4,000 cm^−1^). The NIR spectra of pharmaceutical tablets are shown in Fig. 1c. As seen in the raw spectra, there were many clear absorption bands of the API from 900 to 1,630 nm.

There was also severe spectral overlap and baseline drift in the spectra of corn, Yinhuang granules, and pharmaceutical tablets. Light scattering affected the raw spectra as well; this depended on the physical conditions such as particle size, density and variation among lots.

### Reference method of three spectral datasets

The response variable was the relative content of water in the corn (%, w/w). A detailed description of sample conditions can be found at http://www.eigenvector.com/data/Corn/index.html. The other standard dataset of pharmaceutical tablets included 655 samples. The assay values of the API (%, w/w) were used as a reference value.

Baicalin in Yinhuang granules was quantitated with a calibration curve based on the concentration range from 25.4 to 50.8 μg mL^−1^ of baicalin using consecutive injections of different concentrations. The regression equation was y = 20.25x + 35.10 (r = 1.0000) with y being the peak area in mAUs and x being the concentration of injection (μg mL^−1^). We concluded that high performance liquid chromatography (HPLC) was suitable for quantitation. Baicalin concentrations varied 1.61% to 6.66% (mg mg^−1^). The reference values of baicalin were accurate and could be used in NIR models.

### The selection of sample sets using three datasets

The Kennard-Stone (K-S) algorithm selected the calibration and validation sets. The corn samples included 53 samples for calibration set, and 27 samples for external validation. In addition, the Yinhuang granule dataset used 48 and 24 samples in the calibration and validation sets, respectively. The three different subsets of pharmaceutical tablets were used as the calibration set (155), validation set (460), and test set (40). The statistics for water, baicalin, and API contents in the calibration, validation, and test sets are summarized in Table 1.

### Processing trajectory of PLS model

**Figure 2:**
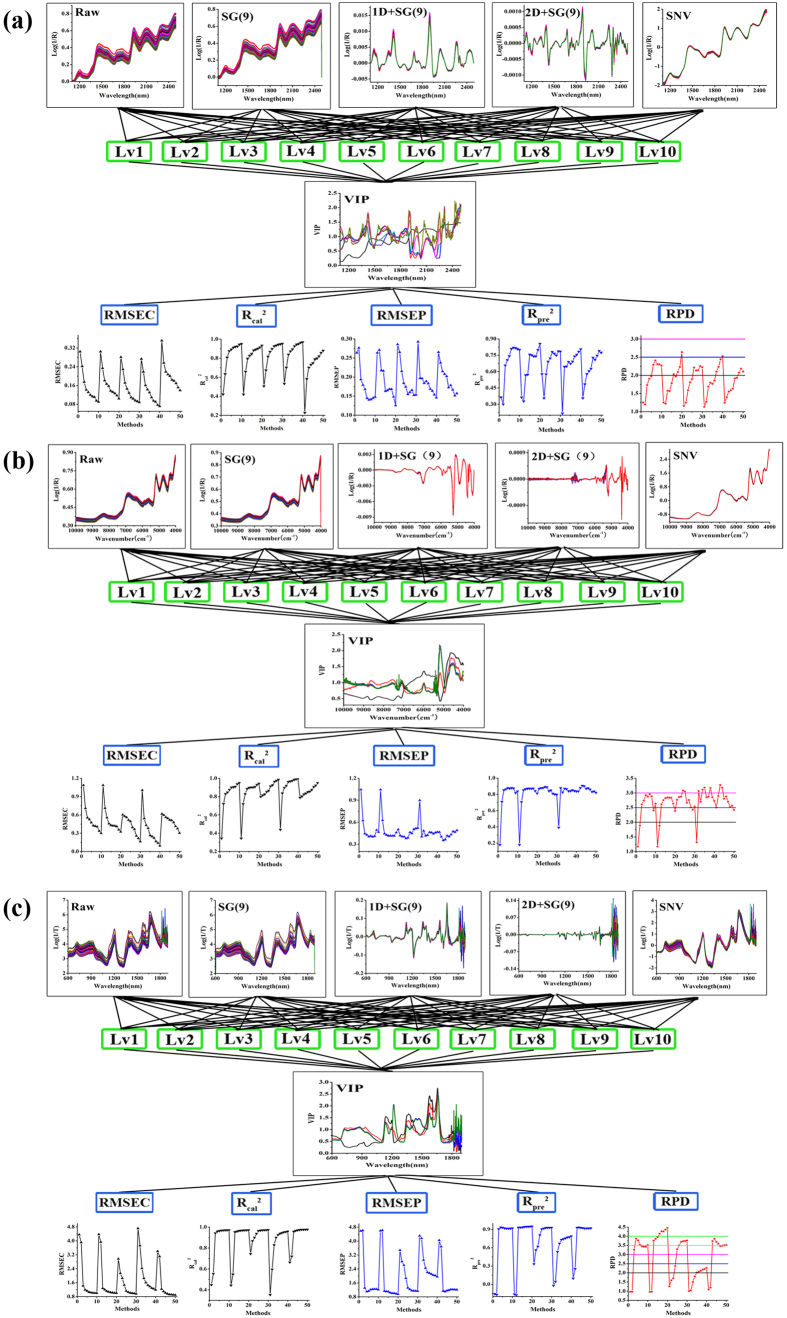
Schematic diagram of processing trajectory and assessment of PLS model corn samples (**a**), Yinhuang granules samples (**b**) and pharmaceutical tablets sample (**c**).

Using the corn dataset as an example, the calibration spectra were preprocessed with different methods including Standard Normal Variate (SNV) and Savitzky-Golay smoothing with 9 points (SG(9)), as well as SG(9) combined with the derivative spectra. The latent factor was set from 1 to 10 to avoid over-fitting. VIP was then used to select variables with different latent factors. Finally, the process routines from PLS model development and validation were selected (Fig. 2). The parameters for PLS models in water, baicalin and API are shown in Table. 1s-3s. There are various trends in the model evaluation indexes. In Fig. 2a, we see that the RMSEC and RMSEP decreased and the R_cal_^2^, R_pre_^2^, and RPD increased with increasing latent factor coupled with different pretreatment methods.

The model for Yinhuang granules dataset is shown in Fig. 2b. This was different from the corn dataset model. The Yinhuang granules dataset model used first derivative spectra (1D) combined with SG(9) and SNV to preprocess the spectra. This was superior to other spectra pretreatment methods in which the various trends of the model evaluation indexes were unclear. The model for pharmaceutical tablets is shown in Fig. 2c. Changes in model evaluation indexes were not obvious when the latent factor increased to a certain value.

**Figure 3:**
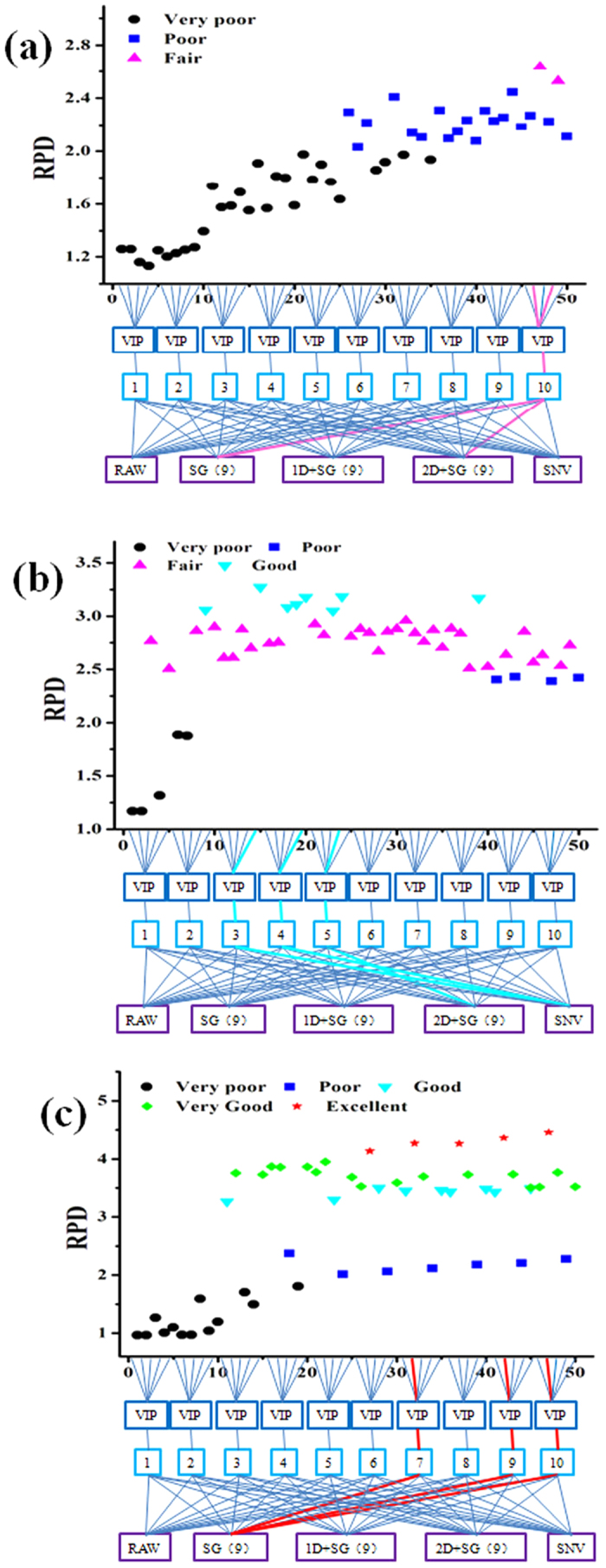
Schematic diagram of processing trajectory of PLS model corn samples (**a**), Yinhuang granules samples (**b**) and pharmaceutical tablets sample (**c**).

However, this result showed that there was more than one modeling path that can ensure a successful model. There were two fair PLS models with RPD between 2.5 to 3 (Fig. 3a) including: 1) a combinational method of SG(9) spectral pretreatment, VIP, and 10 factors, and 2) a combinational method using the second derivative spectra (2D) combined with SG(9) spectral pretreatment, VIP, and 10 factors. Most of PLS models were fair and there were also some good model paths with RPD values greater than 3 (Fig. 3b). In Fig. 3c, there were many models with good performance that adopted a processing trajectory.

In the previous modeling process routine, the parameters included spectral pretreatment methods, variable selection and latent factors were optimized one at a time. Table 4s shows that this is a good approach to path modeling versus step-by-step parameter optimization. The optimal nonsystematic parameters of the water PLS model were the raw spectra and VIP-selecting variables under 7 factors; the model performance was poor. However, processing trajectory showed that two fair model could be obtained through combinational methods using SG(9) pretreatment, VIP, and 10 factors as well as a combinational method of 2D + SG(9) pretreatment, VIP, and 10 factors. The best nonsystematic parameter combination for the baicalin PLS model was SNV pretreatment and VIP-selecting variables under 4 factors. This gave good model performance. However, the processing trajectory showed eight good models with different systematic parameter combinations. The best parameter combination of the baicalin PLS model was a SNV spectra pretreatment with VIP-selecting variables under 3 factors.

The parameter combination for the API model used raw spectra with VIP-selecting variables under 4 factors. These were optimized one by one. The model performance was very good. However, there were 18 very good models and 5 excellent models with RPD values above 4 that were obtained by processing trajectory. The best parameter combination for the PLS models were SG(9) spectra pretreatment and VIP selecting variables under 10 factors.

The best models were obtained through a step-by-step optimization and processing trajectory that was tested with a test set of 40 independent samples. The RMSEP of the two models were 0.7904% and 0.5681%, respectively. This demonstrated that the model obtained through the processing trajectory was better than the model optimized step-by-step. This illustrates that the best systematic optimal model parameters were obtained via the processing trajectory.

### Development and validation of calibration models

The model validity was characterized with RMSEC, RMSEP, R_cal_^2^, R_pre_^2^, and RPD. Taking the calibration model of the corn dataset as an example, Fig. 2a showed that the PLS model of water with SG(9) pretreatment and VIP-selecting variables under 10 factors had the best performance. The RMSEC and R_cal_^2^ of the calibration set were 0.1042% and 0.9321, respectively. The RMSEP was 0.1256% – quite close to RMSEC. The RPD and R_pre_^2^ of the validation set were 2.6387 and 0.8554, respectively. These results demonstrated that the PLS model of water also had a good predictive performance.

Similarly, we developed the PLS model of baicalin with SNV spectral pretreatment and VIP-selecting variables under 3 factors. The RMSEC and R_cal_^2^ of the calibration set were 0.5609% and 0.8250, respectively. The RMSEP, R_pre_^2^ and RPD of the baicalin validation set were 0.3524%, 0.9066, and 3.2723, respectively. The model of API with SG(9) pretreatment and VIP-selecting variables under 10 factors was also established. The RMSEC and R_cal_^2^ of the calibration set were 1.0048% and 0.9706. The RMSEP, R_pre_^2^ and RPD of the API validation set were 0.9581%, 0.9493, and 4.4581, respectively.

**Figure 4:**
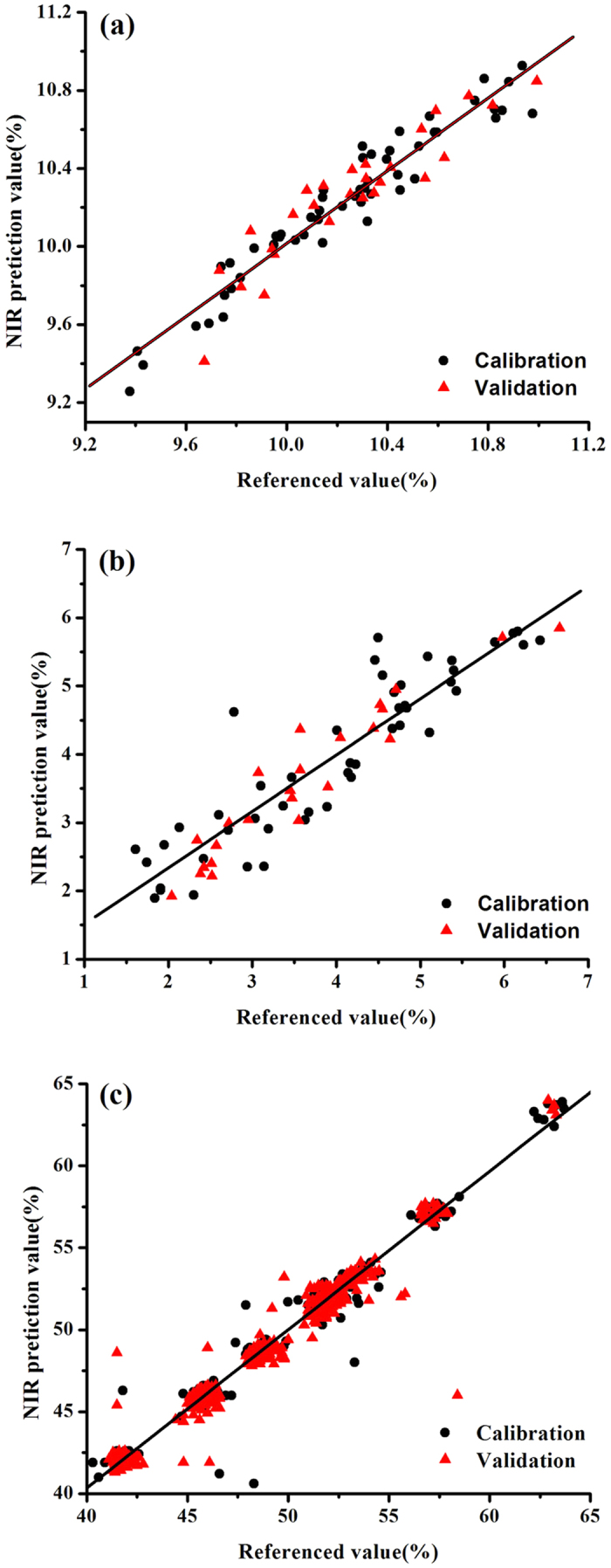
Correlation between the prediction and reference values of corn samples (**a**), Yinhuang granules samples (**b**) and pharmaceutical tablets sample (**c**).

Figure 4 presents data for the PLS models using three datasets. The prediction values were quite close to the wet analysis. The parameters for water, baicalin and API models indicated that NIR could be used for the determination of water, baicalin, and API of corn, Yinhuang granules, and pharmaceutical tablets, respectively.

## Discussion

We proposed the use of processing trajectory to develop and optimize multivariate calibration models. The PLS models were established with different spectral pretreatments and VIP variable selection methods with different latent factors. Based chemometric indicators (RMSEP, RMSEC, R_cal_^2^, R_pre_^2^ and RPD), different PLS models were used to assessed the water, baicalin , and API in corn, Yinhuang granules and pharmaceutical tablets, respectively. The present work demonstrates the feasibility and advantages of processing trajectory in the development and optimization of multivariate calibration models. In conclusion, a systematic procedure for model optimization based on the processing trajectory shows excellent results to develop a robust model. The proposed approach should be integrated into PLS software to improve on the available PLS models.

## Methods

### Datasets

#### Corn spectral dataset

NIR diffuse spectra of corn were obtained from the website as standard data (http://www.eigenvector.com/data/Corn/index.html)^20^. The dataset consists of 80 corn samples. The NIR spectra of corn samples were measured with three spectrometers; the moisture content was included (%, w/w). The spectra were measured with a mp5 NIR spectrometer and the water values were used as reference value. The spectral acquisition range was 1,100–2,498 nm at 2 nm intervals resulting in a total of 700 variables per sample.

#### Yinhuang granules spectral dataset

The dataset consists of diffuse reflectance NIR spectra with 72 Yinhuang granule samples. The response variable was the relative content of active baicalin in the granules (%, w/w). The baicalin content was measured with HPLC as described in the Ch.P. (2010 Edition, Volume I). We used an Agilent 1100 HPLC system (Agilent Technologies, USA) with a vacuum degasser, a quaternary pump, an auto sampler, a thermostatic column compartment, and a diode array detector (DAD). Separation was performed on an ODS column (150 mm, 4.6 mm, 5 mm, Waters, USA) with isocratic elution of the mobile phase consisting of methanol, water and phosphoric acid (50:50:0.2, v/v) at a flow rate of 1.0 mL/min. The column temperature was 30 °C, and the detection wavelength was 274 nm.

The NIR spectra were collected in an integrating sphere diffuse mode with an Antaris Nicolet FT-NIR system (Thermo Fisher Scientific Inc., USA). Each sample spectrum was the result of 32 scans from 10,000 to 4,000 cm^−1^ (a total of 1557 wavenumber variables per sample) at ambient temperature using 8 cm^−1^ resolution. Every sample was scanned three times, and the final spectrum used for each sample was an average of the three results. All NIR spectra were collected and archived using the Thermo Scientific Result software.

#### Pharmaceutical tablets spectral dataset

The dataset for the pharmaceutical tablets was available at http://www.eigenvector.com/data/tablets/index.html. It contains 1,308 spectra of 655 pharmaceutical tablets measured on two similar instruments (Foss/NIR Systems Multitab Spectrometers). The spectral region was from 600 to 1,898 nm with 2 nm increments. The data of each instrument were organized into three different subsets. The assay value of the active ingredient, tablet weight, and tablet hardness were provided. In this work, we used the spectra of the first instrument and the assay values of the API (%, w/w). There were three different sets of 155, 460, and 40 spectra that were used as calibration, validation, and test sets in this study, respectively.

#### Multivariate data analyses

The spectra pretreatment and model development were performed with Unscrambler 9.7 software package (CAMO Software AS, Norway). The VIP-based variable selection methods were implemented with custom routines in MATLAB (MATLAB, The MathWorks, Massachussetts).

#### Summary of the proposed procedure

The procedure we used to track and evaluate modeling processes with different spectral pretreatment methods, latent factors, and VIP variable selection is summarized in Fig. 2 and is detailed in the following section.

Step 1. The modeling parameters and their levels were defined, which contained spectral pretreatment methods, latent factors, and variable selection methods.

Step 2. The evaluation indexes of model were established to include RMSEC, R_cal_^2^, RMSEP, and R_pre_^2^. Important parameters for the RPD were also included to assure the model assessment.

Step 3. The calibration and validation data sets were selected to ensure that both datasets were representative of the experimental design.

Step 4. We ran the PLS models established for all systematic parameters optimization. The results for the evaluation indexes of each model were registered.

Step 5. The results were analyzed, and the trajectory routes for PLS modeling were defined.

Step 6. PLS model was established at the best systematic optimization of parameters. When necessary, the model was further refined to exclude samples with higher spectral and concentration residuals.

Step 7. The model was applied to routine analysis and was continuously monitored to check for needed updates.

#### Multivariate calibration

The PLS was used to build calibration models. First, the K-S algorithm was used to split the data set into calibration and validation sets (2:1) such that the samples could be selected to represent the entire experimental domain. We used spectral pretreatment to decrease baseline shifts and remove the scattering effect created by diffuse reflectance and overlapping peaks. These caused detrimental effects on the signal-to-noise ratio. We used SNV, SG(9), and SG(9) combined with derivative spectra to remedy this. Meanwhile, VIP was utilized to select variables for identifying highly informative features^21^. The performance of the regression models was evaluated in terms of the R_cal_^2^ and RMSEC. The prediction ability of the external validation was assessed by RMSEP, R_pre_^2^ and RPD.

Generally, the optimal number of latent factors was determined from the result of 10-fold cross validation tests after spectral pretreatment. The VIP variable selection algorithms were then used to select the characteristic variables. The ideal methods for spectral pretreatment, the number of latent factors and models were selected based on the RMSEC, RMSEP, R_cal_^2^, R_pre_^2^, RPD, etc. Unlike the previous method, this approach uses the global optimal strategy and trajectory routes of all possible parameter combinations.

## Additional Information

**How to cite this article**: Zhao, N. *et al*. Optimization of Parameter Selection for Partial Least Squares Model Development. *Sci. Rep*. **5**, 11647; doi: 10.1038/srep11647 (2015).

## Supplementary Material

Supplementary Information

## Figures and Tables

**Table 1:** The statistic of water, baicalin and API contents in the calibration and validation sets.

Analyte	sample set	sample number	Min(%)	Max(%)	Mean(%)	SD(%)
corn	calibration	53	9.38	10.98	10.22	0.41
	validation	27	9.67	10.99	10.25	0.34
Yinhuang granules	calibration	48	1.61	6.43	3.94	0.02
	validation	24	2.04	6.66	3.60	1.18
pharmaceutical tablets	calibration	155	40.30	63.70	50.78	5.88
	validation	460	41.10	63.30	49.76	4.26
	test	40	50.20	52.40	51.71	0.54
