# Supplementary material for: Optimization of Parameter Selection for Partial Least Squares Model Development
Source: Sci Rep. 2015 Jul 13;5:11647. doi: 10.1038/srep11647 (PMC4499800; doi:10.1038/srep11647)
Supplement: Supplementary Information [file srep11647-s1.doc]

**Optimisation of Parameter Selection for Partial Least Squares Model Development**

**Na Zhao**, **Zhi-sheng Wu***, **Qiao Zhang, Xinyuan Shi**, **Qun Ma, Yan-jiang Qiao***

**Supplementary Table S1∣The parameters of PLS models of water via** different spectra pretreatment and VIP selecting variables.

| Model | Pretreatment | Latent factor | RMSEC | R_Cal_^2^ | RMSEP | R_Pre_^2^ | RPD | Classification |
| --- | --- | --- | --- | --- | --- | --- | --- | --- |
| 1 | Raw | 1 | 0.3041 | 0.4211 | 0.2636 | 0.3636 | 1.2545 | Very poor |
| 2 | Raw | 2 | 0.2418 | 0.6340 | 0.2763 | 0.3005 | 1.2004 | Very poor |
| 3 | Raw | 3 | 0.1751 | 0.8082 | 0.1935 | 0.6569 | 1.7356 | Very poor |
| 4 | Raw | 4 | 0.1416 | 0.8745 | 0.1790 | 0.7063 | 1.9076 | Very poor |
| 5 | Raw | 5 | 0.1315 | 0.8918 | 0.1677 | 0.7422 | 1.9736 | Very poor |
| 6 | Raw | 6 | 0.1197 | 0.9103 | 0.1445 | 0.8088 | 2.2936 | Poor |
| 7 | Raw | 7 | 0.1134 | 0.9196 | 0.1403 | 0.8196 | 2.4084 | Poor |
| 8 | Raw | 8 | 0.1109 | 0.9230 | 0.1414 | 0.8168 | 2.3062 | Poor |
| 9 | Raw | 9 | 0.0952 | 0.9432 | 0.1448 | 0.8080 | 2.3046 | Poor |
| 10 | Raw | 10 | 0.0901 | 0.9492 | 0.1479 | 0.7995 | 2.2673 | Poor |
| 11 | SG(9) | 1 | 0.3044 | 0.4200 | 0.2635 | 0.3639 | 1.2545 | Very poor |
| 12 | SG(9) | 2 | 0.2335 | 0.6588 | 0.2707 | 0.3285 | 1.2244 | Very poor |
| 13 | SG(9) | 3 | 0.1812 | 0.7945 | 0.2174 | 0.5671 | 1.5756 | Very poor |
| 14 | SG(9) | 4 | 0.1667 | 0.8261 | 0.2160 | 0.5726 | 1.5690 | Very poor |
| 15 | SG(9) | 5 | 0.1572 | 0.8454 | 0.1629 | 0.7568 | 1.7843 | Very poor |
| 16 | SG(9) | 6 | 0.1370 | 0.8825 | 0.1652 | 0.7501 | 2.0295 | Poor |
| 17 | SG(9) | 7 | 0.1322 | 0.8906 | 0.1684 | 0.7403 | 1.9702 | Very poor |
| 18 | SG(9) | 8 | 0.1264 | 0.8999 | 0.1681 | 0.7412 | 2.0926 | Poor |
| 19 | SG(9) | 9 | 0.1175 | 0.9135 | 0.1483 | 0.7986 | 2.2298 | Poor |
| 20 | SG(9) | 10 | 0.1042 | 0.9321 | 0.1256 | 0.8554 | 2.6387 | Fair |
| 21 | 1D+SG(9) | 1 | 0.2808 | 0.5065 | 0.2855 | 0.5768 | 1.1590 | Very poor |
| 22 | 1D+SG(9) | 2 | 0.2286 | 0.6730 | 0.2651 | 0.3564 | 1.2512 | Very poor |
| 23 | 1D+SG(9) | 3 | 0.1711 | 0.8167 | 0.2152 | 0.5758 | 1.5882 | Very poor |
| 24 | 1D+SG(9) | 4 | 0.1456 | 0.8674 | 0.1838 | 0.6909 | 1.8102 | Very poor |
| 25 | 1D+SG(9) | 5 | 0.1281 | 0.8973 | 0.1742 | 0.7220 | 1.8969 | Very poor |
| 26 | 1D+SG(9) | 6 | 0.1090 | 0.9256 | 0.1549 | 0.7802 | 2.2151 | Poor |
| 27 | 1D+SG(9) | 7 | 0.1034 | 0.9331 | 0.1602 | 0.7650 | 2.1351 | Poor |
| 28 | 1D+SG(9) | 8 | 0.0959 | 0.9424 | 0.1656 | 0.7488 | 2.1432 | Poor |
| 29 | 1D+SG(9) | 9 | 0.0924 | 0.9466 | 0.1525 | 0.7869 | 2.2537 | Poor |
| 30 | 1D+SG(9) | 10 | 0.0883 | 0.9512 | 0.1533 | 0.7846 | 2.2239 | Poor |
| 31 | 2D+SG(9) | 1 | 0.2731 | 0.5330 | 0.2925 | 0.2164 | 1.1321 | Very poor |
| 32 | 2D+SG(9) | 2 | 0.2181 | 0.7021 | 0.1971 | 0.6442 | 1.2682 | Very poor |
| 33 | 2D+SG(9) | 3 | 0.1608 | 0.8381 | 0.1961 | 0.6477 | 1.6894 | Very poor |
| 34 | 2D+SG(9) | 4 | 0.1427 | 0.8725 | 0.1840 | 0.6898 | 1.7986 | Very poor |
| 35 | 2D+SG(9) | 5 | 0.1302 | 0.8940 | 0.1888 | 0.6734 | 1.7665 | Very poor |
| 36 | 2D+SG(9) | 6 | 0.1089 | 0.9257 | 0.1831 | 0.6927 | 1.8571 | Very poor |
| 37 | 2D+SG(9) | 7 | 0.0947 | 0.9438 | 0.1684 | 0.7402 | 2.1031 | Poor |
| 38 | 2D+SG(9) | 8 | 0.0902 | 0.9491 | 0.1635 | 0.7552 | 2.2342 | Poor |
| 39 | 2D+SG(9) | 9 | 0.0770 | 0.9629 | 0.1538 | 0.7832 | 2.4434 | Poor |
| 40 | 2D+SG(9) | 10 | 0.0714 | 0.9681 | 0.1459 | 0.8051 | 2.5278 | Fair |
| 41 | SNV | 1 | 0.3511 | 0.2283 | 0.2650 | 0.3565 | 1.2470 | Very poor |
| 42 | SNV | 2 | 0.2563 | 0.5824 | 0.2372 | 0.4847 | 1.3977 | Very poor |
| 43 | SNV | 3 | 0.2184 | 0.7015 | 0.2147 | 0.5779 | 1.5523 | Very poor |
| 44 | SNV | 4 | 0.2072 | 0.7312 | 0.2078 | 0.6046 | 1.5905 | Very poor |
| 45 | SNV | 5 | 0.2004 | 0.7487 | 0.2018 | 0.6267 | 1.6368 | Very poor |
| 46 | SNV | 6 | 0.1839 | 0.7884 | 0.1725 | 0.7273 | 1.9163 | Very poor |
| 47 | SNV | 7 | 0.1796 | 0.7981 | 0.1782 | 0.7092 | 1.9351 | Very poor |
| 48 | SNV | 8 | 0.1695 | 0.8203 | 0.1664 | 0.7464 | 2.0758 | Poor |
| 49 | SNV | 9 | 0.1515 | 0.8563 | 0.1514 | 0.7900 | 2.1821 | Poor |
| 50 | SNV | 10 | 0.1392 | 0.8788 | 0.1569 | 0.7744 | 2.1070 | Poor |

***The unit of RMSEC and RMSEP is mg/mg %).**

**Supplementary Table S2∣The parameters of PLS models of bacalin via different spectra pretreatment and VIP** selecting variables.

| Model | Pretreatment | Latent factor | RMSEC | R_Cal_^2^ | RMSEP | R_Pre_^2^ | RPD | Classification |
| --- | --- | --- | --- | --- | --- | --- | --- | --- |
| 1 | Raw | 1 | 1.0856 | 0.3442 | 1.0444 | 0.1794 | 1.1684 | Very poor |
| 2 | Raw | 2 | 0.7080 | 0.7211 | 0.6222 | 0.7087 | 1.8843 | Very poor |
| 3 | Raw | 3 | 0.5532 | 0.8297 | 0.4442 | 0.8515 | 2.6067 | Fair |
| 4 | Raw | 4 | 0.5252 | 0.8465 | 0.4262 | 0.8633 | 2.7420 | Fair |
| 5 | Raw | 5 | 0.4738 | 0.8751 | 0.4035 | 0.8775 | 2.9231 | Fair |
| 6 | Raw | 6 | 0.4239 | 0.9000 | 0.4126 | 0.8719 | 2.8781 | Fair |
| 7 | Raw | 7 | 0.4162 | 0.9036 | 0.4009 | 0.8791 | 2.9568 | Fair |
| 8 | Raw | 8 | 0.4050 | 0.9087 | 0.4100 | 0.8735 | 2.8816 | Fair |
| 9 | Raw | 9 | 0.3361 | 0.9372 | 0.4926 | 0.8174 | 2.4040 | Poor |
| 10 | Raw | 10 | 0.2998 | 0.9500 | 0.4656 | 0.8369 | 2.6335 | Fair |
| 11 | SG(9) | 1 | 1.0856 | 0.3442 | 1.0443 | 0.1796 | 1.1685 | Very poor |
| 12 | SG(9) | 2 | 0.7103 | 0.7193 | 0.6251 | 0.7060 | 1.8762 | Very poor |
| 13 | SG(9) | 3 | 0.5546 | 0.8289 | 0.4438 | 0.8518 | 2.6084 | Fair |
| 14 | SG(9) | 4 | 0.4560 | 0.8843 | 0.4258 | 0.8636 | 2.7523 | Fair |
| 15 | SG(9) | 5 | 0.4314 | 0.8965 | 0.4144 | 0.8708 | 2.8208 | Fair |
| 16 | SG(9) | 6 | 0.4214 | 0.9012 | 0.4133 | 0.8715 | 2.8413 | Fair |
| 17 | SG(9) | 7 | 0.4209 | 0.9014 | 0.4139 | 0.8711 | 2.8406 | Fair |
| 18 | SG(9) | 8 | 0.4095 | 0.9067 | 0.4133 | 0.8715 | 2.8358 | Fair |
| 19 | SG(9) | 9 | 0.3566 | 0.9292 | 0.4590 | 0.8415 | 2.6370 | Fair |
| 20 | SG(9) | 10 | 0.3234 | 0.9418 | 0.4998 | 0.8120 | 2.3879 | Poor |
| 21 | 1D+SG(9) | 1 | 0.6019 | 0.7985 | 0.4166 | 0.8694 | 2.7672 | Fair |
| 22 | 1D+SG(9) | 2 | 0.5781 | 0.8141 | 0.4042 | 0.8771 | 2.8592 | Fair |
| 23 | 1D+SG(9) | 3 | 0.5597 | 0.8257 | 0.4012 | 0.8789 | 2.8738 | Fair |
| 24 | 1D+SG(9) | 4 | 0.5157 | 0.8520 | 0.3799 | 0.8914 | 3.0822 | Good |
| 25 | 1D+SG(9) | 5 | 0.4924 | 0.8651 | 0.3872 | 0.8872 | 3.0502 | Good |
| 26 | 1D+SG(9) | 6 | 0.3950 | 0.9132 | 0.4610 | 0.8401 | 2.6675 | Fair |
| 27 | 1D+SG(9) | 7 | 0.3639 | 0.9263 | 0.4469 | 0.8497 | 2.7587 | Fair |
| 28 | 1D+SG(9) | 8 | 0.2563 | 0.9634 | 0.5032 | 0.8095 | 2.5089 | Fair |
| 29 | 1D+SG(9) | 9 | 0.2165 | 0.9739 | 0.5145 | 0.8009 | 2.4303 | Poor |
| 30 | 1D+SG(9) | 10 | 0.1626 | 0.9853 | 0.5206 | 0.7961 | 2.5327 | Fair |
| 31 | 2D+SG(9) | 1 | 1.0045 | 0.4386 | 0.8993 | 0.3916 | 1.3167 | Very poor |
| 32 | 2D+SG(9) | 2 | 0.5414 | 0.8369 | 0.4022 | 0.8783 | 3.0590 | Good |
| 33 | 2D+SG(9) | 3 | 0.4301 | 0.8971 | 0.4815 | 0.8256 | 2.6987 | Fair |
| 34 | 2D+SG(9) | 4 | 0.3818 | 0.9189 | 0.4157 | 0.8700 | 3.1107 | Good |
| 35 | 2D+SG(9) | 5 | 0.2640 | 0.9612 | 0.4232 | 0.8653 | 3.1838 | Good |
| 36 | 2D+SG(9) | 6 | 0.2457 | 0.9664 | 0.4624 | 0.8391 | 2.8530 | Fair |
| 37 | 2D+SG(9) | 7 | 0.2220 | 0.9726 | 0.4594 | 0.8413 | 2.8689 | Fair |
| 38 | 2D+SG(9) | 8 | 0.1661 | 0.9847 | 0.4444 | 0.8514 | 3.1693 | Good |
| 39 | 2D+SG(9) | 9 | 0.1377 | 0.9895 | 0.4575 | 0.8426 | 2.8536 | Fair |
| 40 | 2D+SG(9) | 10 | 0.0940 | 0.9951 | 0.4657 | 0.8368 | 2.7255 | Fair |
| 41 | SNV | 1 | 0.6156 | 0.7891 | 0.4641 | 0.8379 | 2.5036 | Fair |
| 42 | SNV | 2 | 0.5963 | 0.8021 | 0.4008 | 0.8792 | 2.8957 | Fair |
| 43 | SNV | 3 | 0.5609 | 0.8250 | 0.3524 | 0.9066 | 3.2723 | Good |
| 44 | SNV | 4 | 0.5430 | 0.8356 | 0.3633 | 0.9007 | 3.1787 | Good |
| 45 | SNV | 5 | 0.5180 | 0.8507 | 0.4153 | 0.8702 | 2.8061 | Fair |
| 46 | SNV | 6 | 0.5096 | 0.8555 | 0.4041 | 0.8771 | 2.8788 | Fair |
| 47 | SNV | 7 | 0.4763 | 0.8738 | 0.4307 | 0.8604 | 2.7036 | Fair |
| 48 | SNV | 8 | 0.4132 | 0.9050 | 0.4738 | 0.8311 | 2.5229 | Fair |
| 49 | SNV | 9 | 0.3750 | 0.9218 | 0.4655 | 0.8369 | 2.5647 | Fair |
| 50 | SNV | 10 | 0.3023 | 0.9492 | 0.4881 | 0.8207 | 2.4213 | Poor |

***The unit of RMSEC and RMSEP is mg/mg (%).**

**Supplementary Table S3∣**The parameters of PLS models of API via different spectra pretreatment and VIP selecting variables.

| Model | Preteatment | Latent factor | RMSEC | R_Cal_^2^ | RMSEP | R_Pre_^2^ | RPD | Classification |
| --- | --- | --- | --- | --- | --- | --- | --- | --- |
| 1 | Raw | 1 | 4.3551 | 0.4470 | 4.5815 | -0.1583 | 0.9658 | Very poor |
| 2 | Raw | 2 | 3.9090 | 0.5544 | 4.6054 | -0.1704 | 0.9713 | Very poor |
| 3 | Raw | 3 | 1.4212 | 0.9411 | 1.3083 | 0.9056 | 3.2647 | Good |
| 4 | Raw | 4 | 1.1926 | 0.9585 | 1.1024 | 0.9329 | 3.8689 | Very good |
| 5 | Raw | 5 | 1.1274 | 0.9629 | 1.1328 | 0.9292 | 3.7720 | Very good |
| 6 | Raw | 6 | 1.0601 | 0.9672 | 1.2144 | 0.9186 | 3.5267 | Very good |
| 7 | Raw | 7 | 1.0348 | 0.9688 | 1.2415 | 0.9150 | 3.4488 | Good |
| 8 | Raw | 8 | 1.0165 | 0.9699 | 1.2461 | 0.9143 | 3.4327 | Good |
| 9 | Raw | 9 | 1.0067 | 0.9704 | 1.2458 | 0.9144 | 3.4319 | Good |
| 10 | Raw | 10 | 0.9980 | 0.9710 | 1.2148 | 0.9186 | 3.5171 | Very good |
| 11 | SG(9) | 1 | 4.3708 | 0.4429 | 4.5897 | -0.1624 | 0.9641 | Very poor |
| 12 | SG(9) | 2 | 3.9330 | 0.5490 | 4.6180 | -0.1768 | 0.9687 | Very poor |
| 13 | SG(9) | 3 | 1.2774 | 0.9524 | 1.1341 | 0.9290 | 3.7544 | Very good |
| 14 | SG(9) | 4 | 1.2017 | 0.9578 | 1.1070 | 0.9324 | 3.8563 | Very good |
| 15 | SG(9) | 5 | 1.1187 | 0.9635 | 1.1010 | 0.9331 | 3.9518 | Very good |
| 16 | SG(9) | 6 | 1.0759 | 0.9662 | 1.0381 | 0.9405 | 4.1358 | Excellent |
| 17 | SG(9) | 7 | 1.0451 | 0.9682 | 1.0064 | 0.9441 | 4.2679 | Excellent |
| 18 | SG(9) | 8 | 1.0262 | 0.9693 | 1.0055 | 0.9442 | 4.2641 | Excellent |
| 19 | SG(9) | 9 | 1.0109 | 0.9702 | 0.9804 | 0.9470 | 4.3627 | Excellent |
| 20 | SG(9) | 10 | 1.0048 | 0.9706 | 0.9581 | 0.9493 | 4.4581 | Excellent |
| 21 | 1D+SG(9) | 1 | 2.9653 | 0.7436 | 3.4772 | 0.3328 | 1.2631 | Very poor |
| 22 | 1D+SG(9) | 2 | 2.1388 | 0.8667 | 2.8237 | 0.5600 | 1.5926 | Very poor |
| 23 | 1D+SG(9) | 3 | 1.8544 | 0.8997 | 2.6156 | 0.6196 | 1.7064 | Very poor |
| 24 | 1D+SG(9) | 4 | 1.4648 | 0.9374 | 1.8716 | 0.8067 | 2.3721 | Poor |
| 25 | 1D+SG(9) | 5 | 1.1241 | 0.9632 | 1.3213 | 0.9037 | 3.2981 | Good |
| 26 | 1D+SG(9) | 6 | 1.0563 | 0.9675 | 1.2335 | 0.9160 | 3.4989 | Good |
| 27 | 1D+SG(9) | 7 | 1.0166 | 0.9699 | 1.1582 | 0.9260 | 3.6958 | Very good |
| 28 | 1D+SG(9) | 8 | 0.9960 | 0.9711 | 1.1459 | 0.9275 | 3.7295 | Very good |
| 29 | 1D+SG(9) | 9 | 0.9866 | 0.9716 | 1.1436 | 0.9278 | 3.7370 | Very good |
| 30 | 1D+SG(9) | 10 | 0.9616 | 0.9730 | 1.1311 | 0.9294 | 3.7684 | Very good |
| 31 | 2D+SG(9) | 1 | 4.7109 | 0.3529 | 4.3003 | -0.0204 | 1.0090 | Very poor |
| 32 | 2D+SG(9) | 2 | 3.7243 | 0.5956 | 4.1582 | 0.0459 | 1.0411 | Very poor |
| 33 | 2D+SG(9) | 3 | 2.4267 | 0.8283 | 2.9139 | 0.5315 | 1.4958 | Very poor |
| 34 | 2D+SG(9) | 4 | 1.8518 | 0.9000 | 2.4520 | 0.6682 | 1.8063 | Very poor |
| 35 | 2D+SG(9) | 5 | 1.6050 | 0.9249 | 2.2061 | 0.7314 | 2.0132 | Poor |
| 36 | 2D+SG(9) | 6 | 1.4624 | 0.9376 | 2.1640 | 0.7416 | 2.0621 | Poor |
| 37 | 2D+SG(9) | 7 | 1.3817 | 0.9443 | 2.1072 | 0.7550 | 2.1138 | Poor |
| 38 | 2D+SG(9) | 8 | 1.2939 | 0.9512 | 2.0467 | 0.7688 | 2.1754 | Poor |
| 39 | 2D+SG(9) | 9 | 1.2540 | 0.9541 | 2.0206 | 0.7747 | 2.2061 | Poor |
| 40 | 2D+SG(9) | 10 | 1.1888 | 0.9588 | 1.9557 | 0.7889 | 2.2724 | Poor |
| 41 | SNV | 1 | 3.3991 | 0.6631 | 4.0442 | 0.0975 | 1.0984 | Very poor |
| 42 | SNV | 2 | 3.1006 | 0.7197 | 3.6766 | 0.2541 | 1.1953 | Very poor |
| 43 | SNV | 3 | 1.2301 | 0.9559 | 1.1420 | 0.9280 | 3.7298 | Very good |
| 44 | SNV | 4 | 1.1001 | 0.9647 | 1.1059 | 0.9325 | 3.8609 | Very good |
| 45 | SNV | 5 | 1.0172 | 0.9698 | 1.1595 | 0.9258 | 3.6885 | Very good |
| 46 | SNV | 6 | 0.9624 | 0.9730 | 1.1881 | 0.9221 | 3.5915 | Very good |
| 47 | SNV | 7 | 0.9263 | 0.9750 | 1.2319 | 0.9163 | 3.4595 | Good |
| 48 | SNV | 8 | 0.9160 | 0.9755 | 1.2231 | 0.9174 | 3.4846 | Good |
| 49 | SNV | 9 | 0.9030 | 0.9762 | 1.2158 | 0.9184 | 3.5064 | Very good |
| 50 | SNV | 10 | 0.8935 | 0.9767 | 1.2105 | 0.9191 | 3.5189 | Very good |

***The unit of RMSEC and RMSEP is mg/mg (%).**

**Supplementary Table S4∣The parameters of PLS models of water and bacalin** via different spectra pretreatment and VIP selecting variables.

| Analyte | Pretreatment | Latent factors | RMSEC | R_cal_^2^ | RMSEP | R_pre_^2^ | RPD | Classification |
| --- | --- | --- | --- | --- | --- | --- | --- | --- |
| corn | Raw | 7 | 0.1134 | 0.9196 | 0.1403 | 0.8196 | 2.4084 | Poor |
|  | SG(9) | 9 | 0.1175 | 0.9135 | 0.1483 | 0.7986 | 2.2298 | Poor |
|  | 1D+SG(9) | 7 | 0.1034 | 0.9331 | 0.1602 | 0.7650 | 2.1351 | Poor |
|  | 2D+SG(9) | 5 | 0.1302 | 0.8940 | 0.1888 | 0.6734 | 1.7665 | Very poor |
|  | SNV | 6 | 0.1839 | 0.7884 | 0.1725 | 0.7273 | 1.9163 | Very poor |
| yinghuang | Raw | 5 | 0.4738 | 0.8751 | 0.4035 | 0.8775 | 2.9231 | Fair |
|  | SG(9) | 5 | 0.4314 | 0.8965 | 0.4144 | 0.8708 | 2.8208 | Fair |
|  | 1D+SG(9) | 5 | 0.4924 | 0.8651 | 0.3872 | 0.8872 | 3.0502 | Good |
|  | 2D+SG(9) | 3 | 0.4301 | 0.8971 | 0.4815 | 0.8256 | 2.6987 | Fair |
|  | SNV | 4 | 0.5430 | 0.8356 | 0.3633 | 0.9007 | 3.1787 | Good |
| pharmaceutical tablets | Raw | 4 | 1.1926 | 0.9585 | 1.1024 | 0.9329 | 3.8689 | Very good |
|  | SG(9) | 4 | 1.2017 | 0.9578 | 1.1070 | 0.9324 | 3.8563 | Very good |
|  | 1D+SG(9) | 4 | 1.4648 | 0.9374 | 1.8716 | 0.8067 | 2.3721 | Poor |
|  | 2D+SG(9) | 6 | 1.4624 | 0.9376 | 2.1640 | 0.7416 | 2.0621 | Poor |
|  | SNV | 3 | 1.2301 | 0.9559 | 1.1420 | 0.9280 | 3.7298 | Very good |

***The unit of RMSEC and RMSEP is mg/mg (%).**
